# The Associations of Serum Uric Acid with Obesity-Related *Acanthosis nigricans* and Related Metabolic Indices

**DOI:** 10.1155/2017/5438157

**Published:** 2017-03-07

**Authors:** Cuiling Zhu, Ran Cui, Mingming Gao, Sharvan Rampersad, Hui You, Chunjun Sheng, Peng Yang, Hui Sheng, Xiaoyun Cheng, Le Bu, Shen Qu

**Affiliations:** ^1^Department of Endocrinology and Metabolism, Shanghai Tenth People's Hospital, Tongji University School of Medicine, Shanghai, China; ^2^Department of Pharmaceutical and Biomedical Sciences, University of Georgia College of Pharmacy, Athens, GA, USA

## Abstract

*Objective*. Recent studies have shown that hyperuricemia (HUA) is associated with hypertension, dyslipidemia, insulin resistance, and metabolic syndrome (MetS). We aimed to examine the relationship of serum UA with *Acanthosis nigricans* (AN) and related metabolic indices in obese patients. *Methods.* A cross-sectional study with 411 obese patients recruited from our department was analyzed in this study. Weight, body mass index (BMI), UA, lipid profile, liver function, and renal function were measured in all participants. Oral glucose tolerance tests were performed, and serum glucose, insulin, and C peptide were measured at 0, 30, 60, 120, and 180 min. *Results.* AN group had higher serum UA levels than OB group. Circulating UA levels were associated with BMI, dyslipidemia, hypertension, IR, and AN. In logistic regression analyses (multivariable‐adjusted), a high serum UA level was associated with high odds ratios (ORs) (95% confidence interval [CI]) for AN in females (ORs = 3.00 and 95% CI [1.02–8.84]) and males (ORs = 6.07 and 95% CI [2.16–17.06]) in the highest quartile (Q4) of serum UA. *Conclusions.* Serum UA levels were positively associated with multiple metabolic abnormalities including obesity, hypertension, hyperglycemia, hyperlipidemia, and AN and may be an important risk factor in the development of AN; further evidences in vitro and in vivo are needed to investigate the direct or indirect relationship.

## 1. Introduction

Uric acid (UA) is the end-product of purine metabolism in humans [1]. In the last few decades, the prevalence of hyperuricemia (HUA) has been rapidly increasing worldwide [2, 3]. HUA has been traditionally considered to be a risk factor for hypertension, diabetes mellitus, cardiovascular disease, renal disease, and metabolic syndrome (MetS) [4–7]. Growing epidemiological studies suggested that serum UA levels may predict the development of MetS. In the study by Lin et al., serum UA levels were elevated significantly as the number of metabolic components increased [8]. In a study in Chinese, the prevalence of MetS increased with rise in serum UA levels and MetS component number presented a significantly increasing trend across serum UA quartiles in both sexes. Additionally, participants with HUA or higher serum UA levels were at significantly higher ORs for MetS and its related components including abdominal obesity, hypertension, hyperglycemia, and low HDL cholesterol [9, 10]. These previous studies suggested that serum UA levels may be a useful predictor for metabolic disorders.

AN is a typical skin lesion characterized by velvety, brownish-black, papillose thickening hyperpigmentation of the skin of the epidermis [11–13]. Clinical studies have shown that obesity-related AN is usually accompanied by metabolic disorders, including overweight, abnormal glucose metabolism, dyslipidemia, and fatty liver [12, 14]. The occurrence of obesity-related AN is significantly related with insulin resistance and hyperinsulinemia [15, 16]. However, in some cases, patients such as obesity or type 2 diabetes with significant insulin resistance do not have AN. This indicates that insulin resistance is not the only predominant factor in the pathophysiological process of AN.

AN, as a disorganized metabolic state, might be predicted by serum UA. However, the role of serum UA level in the development of obesity-related AN is not yet understood. The present study aimed to investigate the relationship of serum UA, AN, and related metabolic indices in obese patients.

## 2. Methods

### 2.1. Study Design

We conducted a cross-sectional study with 411 obese patients recruited from our outpatient and inpatient department from July 2015 to March 2016. They are divided into two groups including 220 obese patients without AN (OB group) and 191 obese patients with AN (AN group). The study protocol was approved by the Hospital Research Ethics Review Committee, and written informed consent was obtained from all participants (Clinical Trials Registration Number is ChiCTROCS-12002381, http://www.who.int/ictrp).

### 2.2. Study Subjects

A total of 411 obesity participants (52.0% females and 48.0% males) were consecutively enrolled in this study. Inclusion criteria: obesity in this study was defined as BMI ≥ 28 kg/m^2^ according to the diagnostic criteria for obesity in a Chinese population [17]. Exclusion criteria included the presence of malignant tumor, renal dysfunction, severe liver dysfunction (aspartate aminotransferase (AST) or alanine aminotransferase (ALT) levels more than 2.5 times the normal value), and a history of preexisting heart disease. In addition, those who were treated using any medication or other therapeutic methods that could influence the weight, glucose metabolism, lipid metabolism, or uric acid levels, such as hypoglycemic agents, lipid-lowering, and uric acid-lowering agents (allopurinol or benzbromarone) in the 3-month period prior to this study had been excluded in this study.

### 2.3. Definitions of *Acanthosis nigricans*

AN is an easily identifiable skin condition that is strongly associated with insulin resistance, characterized by velvety, brownish-black pigmentation of the skin folds, mainly found in the posterior aspect of the neck, axillae, elbows, knees, umbilicus, and occasionally mucosal surfaces [11–15]. A quantitative scale of AN has been developed by Burke et al. [14], 0—absent: not detectable on close inspection; 1—present: clearly present on close visual inspection, not visible to the casual observer, extent not measurable; 2—mild: limited to the base of the skull, does not extend to the lateral margins of the neck (usually <3 inches in breadth); 3—moderate: extending to the lateral margins of the neck (posterior border of the sternocleidomastoid, usually 3–6 inches), should not be visible when the participant is viewed from the front; 4—severe: extending anteriorly (>6 inches), visible when the participant is viewed from the front.

### 2.4. Measurements

All the patients underwent a physical examination (including measurements of height, weight, waist circumference (WC), hip circumference (HC), systolic blood pressure (SBP), diastolic blood pressure (DBP), and percentage of body fat (%)). Signs of *Acanthosis nigricans* were assessed by a trained physician. Routine blood biochemical tests, including serum UA, blood creatinine (Cr), liver function, blood glucose, and blood lipids, were performed. The liver function tests included aspartate aminotransferase (AST) and alanine aminotransferase (ALT). The blood lipid tests included total cholesterol (TC), triglycerides (TG), high-density lipoprotein (HDL), and low-density lipoprotein (LDL). The oral glucose tolerance tests were performed, and the insulin and C peptide levels were measured at 0, 30, 60, 120, and 180 min, among which height and weight were measured by a simple anthropometric measuring instrument (Omron HBF-358, Japan) with patients lightly clothed and without shoes in a standing position. Body fat (%) was measured by dual DEXA. SBP and DBP were measured twice in the right arm of subjects who had been resting for at least 10 min in a seated position using a mercury sphygmomanometer, and BMI was calculated for all participants. WC and HC were made using an unstretched tape without any pressure to the body surface. Blood samples were taken from each subject after an overnight fast. The participants were divided into four quartiles (Q1, Q2, Q3, and Q4) according to the serum UA levels and gender.

BMI was calculated as weight in kilograms divided by the square of height in meters. Insulin resistance was estimated using the homeostasis model assessment of insulin resistance (HOMA-IR) [18], which was calculated as fasting plasma glucose (FPG) (mmol/L) × fasting insulin (FINS) (mU/L)/22.5. Insulin sensitivity index (ISI) was calculated as 1/FINS (mU/L) × FPG (mmol/L), which was used to assess the insulin sensitivity.

### 2.5. Statistical Analysis

All statistical analyses were performed using the SPSS 20.0 package. Means and standard deviations (mean ± SD) or medians (interquartile range) were calculated for continuous variables. Non-normally distributed data were logarithmically transformed to normality (HOMA-IR), when needed. Comparisons between groups were tested using Student's *t*-test or analysis of variance (ANOVA), and least significant differences (LSD) post hoc tests, when the data is normally distributed. Non-normally distributed data were analyzed by nonparametric test. Linear regression analyses were used to estimate the trends of continuous variables, and chi-square tests for trends in proportions were performed for categorical variables across the increasing subgroup-specific quartiles of serum UA. Repeated measure ANOVA was used for comparing glucose, insulin, and C peptide level at 0, 30, 60, 120, and 180 min among the four UA groups. Analyses of covariance were performed to estimate the associations between serum UA, HOMA-IR, and ISI in unadjusted and multivariable-adjusted models. To examine associations between serum UA level and AN, we ran three logistic regression models: (1) for age and BMI; (2) for age, BMI, FPG, and LDL; and (3) for HOMA-IR in addition to all covariates in (2) both in females and males. ORs and corresponding 95% CI were calculated. Figures in this study were produced by GraphPad Prism 5 project. All reported *P* values are two-sided and considered statistically significant at <0.05.

## 3. Results

### 3.1. General Characteristics

The clinical and metabolic characteristics of the study population are presented in Table 1. Patients in AN group have more severe metabolic disorders including obesity, hypertension, glucose metabolic disorder, hyperlipidemia, and insulin resistance. The serum UA levels in AN group were found to significantly increase compared with OB group in both men and women. Similarly, weight, BMI, WC, SBP, ALT, and AST were significantly higher in AN group than OB group for both sexes. In addition, height, WHR, DBP, and percentage of body fat increased significantly in females. As for lipid metabolism aspect, the HDL levels in AN group were found to significantly decrease in males, while no significant difference in the levels of TC, TG, and LDL was observed compared with OB group. As for glucose metabolism, there was no significant difference at 0 min glucose levels in either gender. However, patients in AN group showed significantly higher insulin and C peptide levels at 0 min compared with the OB group among females and males. Furthermore, the intransformed HOMA-IR levels significantly increased in AN group for all participants.

### 3.2. Relationship between Serum UA and Metabolic Indices

**Figure 1:**
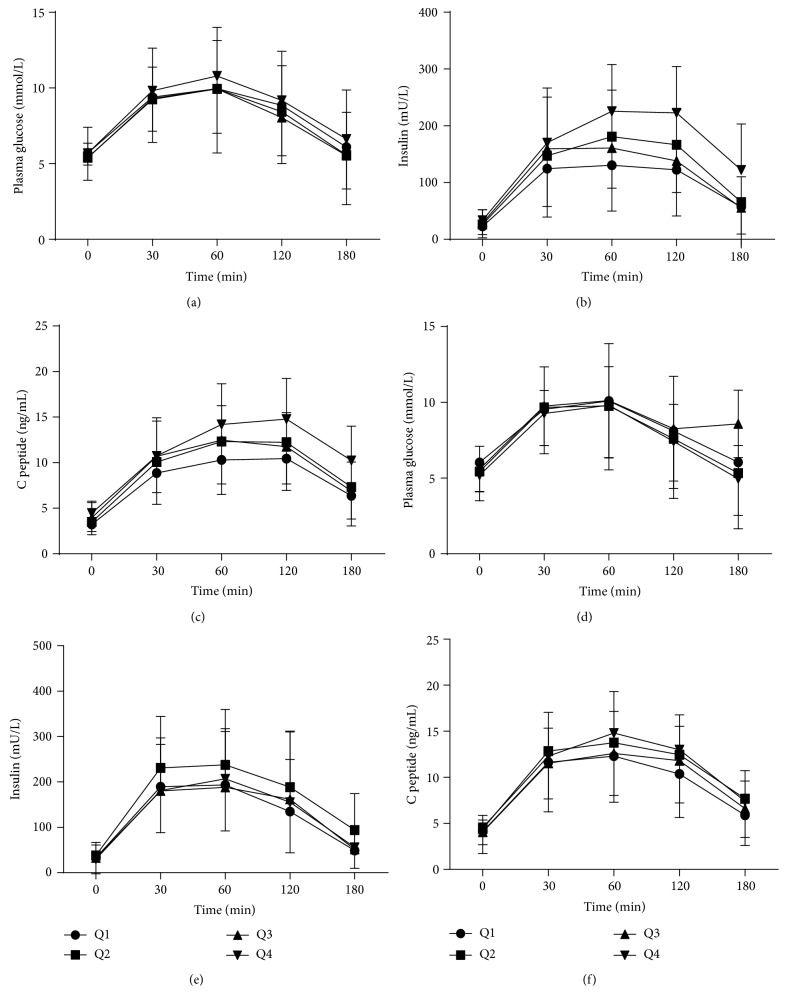
The change of glucose, insulin, and C peptide levels according to serum UA quartiles in females and males. (a) For females, no significant difference was observed in glucose level across quartiles at any point (*P* = 0.320). (b) For females, significant difference was observed in insulin levels across the serum UA quartiles (*P* < 0.001). (c) For females, significant difference was observed in C peptide levels across the serum UA quartiles (*P* < 0.001). (d) For males, no significant difference was observed in glucose level across quartiles at any point (*P* = 0.496). (e) For males, no significant difference was observed in insulin levels across quartiles at any point (*P* = 0.108). (f) For males, significant difference was observed in C peptide levels across the serum UA quartiles (*P* = 0.042).

**Figure 2:**
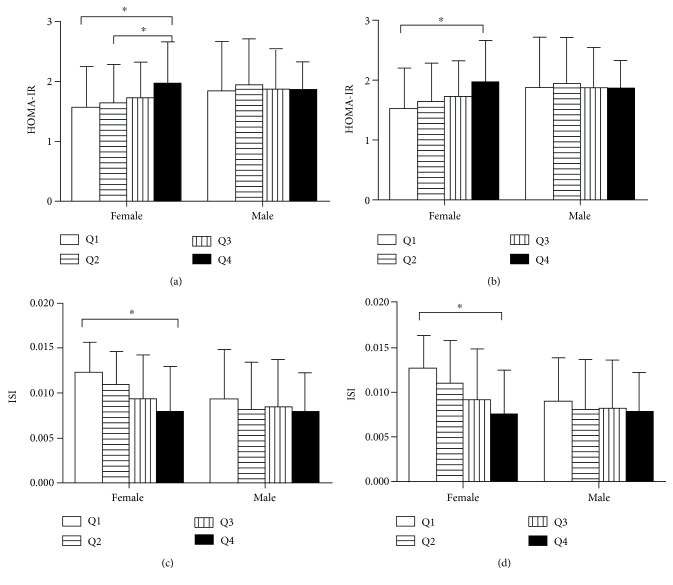
Unadjusted and adjusted HOMA-IR and ISI level in each quartile of serum UA (Q1, Q2, Q3, and Q4) in male and female participants. (a) Unadjusted HOMA-IR was found to significantly increase across the quartiles in females while no significant difference was observed in males. (b) After adjusting for age, SBP, DBP, AST, and WHR, the HOMA-IR levels significantly increased across the quartiles in females while no significant difference was observed in males. (c) Unadjusted ISI was found to significantly decrease across the quartiles in females while no significant difference was observed in males. (d) After adjusting for the abovementioned factors, ISI levels significantly decreased across the quartiles in females while no significant difference was observed in males. ^∗^*P* < 0.05.

Due to the significant difference in serum UA concentrations between females and males, we divided subjects into four gender-specific quartiles (Q1, Q2, Q3, and Q4) according to the serum UA levels as follows: among females, Q1, <310 *μ*mol/L; Q2, 311~360 *μ*mol/L; Q3, 361~424 *μ*mol/L; Q4, ≥425 *μ*mol/L; among males, Q1, <393 *μ*mol/L; Q2, 394~458 *μ*mol/L; Q3, 459~528 *μ*mol/L; Q4, ≥529 *μ*mol/L. We analyzed the mean value of each characteristic and metabolic index for each serum UA quartile (Table 2). We found that BMI, WC, and HC significantly increased from the lowest quartile of serum UA (Q1) to the highest serum UA quartile (Q4) for both sexes. As for blood pressure and lipid profiles, there was different demonstration in different genders. In females, SBP and DBP had significantly positive association with serum UA levels, while, in males, SBP had a significantly positive association with serum UA levels, but DBP had a nonsignificant association with serum UA. In the items of lipid profiles, the levels of TC, TG, and LDL were positively related with serum UA, while HDL levels were negatively related with serum UA in females. In the males, the similar relationship between HDL and UA was observed, while the relationship between other lipid profiles and UA was not significant. As for glucose metabolism (shown in Figure 1), no significant difference was observed in glucose levels between quartiles at any point in either gender (Figures 1(a) and 1(d)). However, among females, significant difference was observed in insulin and C peptide levels across the serum UA quartiles (*P* < 0.001, *P* < 0.001) (Figures 1(b) and 1(c)). Similarly, among males, significant difference was observed in C peptide levels across the serum UA quartiles (*P* = 0.042) (Figure 1(f)), whereas no significant difference was observed in insulin levels across the quartiles (Figure 1(e)). As was shown in Figure 2, before adjusting for potential confounders, HOMA-IR index was found to significantly increase across the serum UA quartiles in females, while no increasing trend was observed in males (Figure 2(a)). Similarly, ISI was found to significantly decrease across the serum UA quartiles in females, and no decreasing trend was observed in males (Figure 2(c)). An analysis of covariance was performed to investigate the association between serum UA levels and HOMA-IR and ISI after controlling for confounders. After adjusting age, SBP, DBP, AST, and WHR, the HOMA-IR was found to have a significantly increasing trend and ISI have a significantly decreasing trend across the quartiles in females while no significant difference across the quartiles was observed in males (Figures 2(b) and 2(d)).

### 3.3. Relationship of Serum UA Levels with the Occurrence of AN

**Figure 3:**
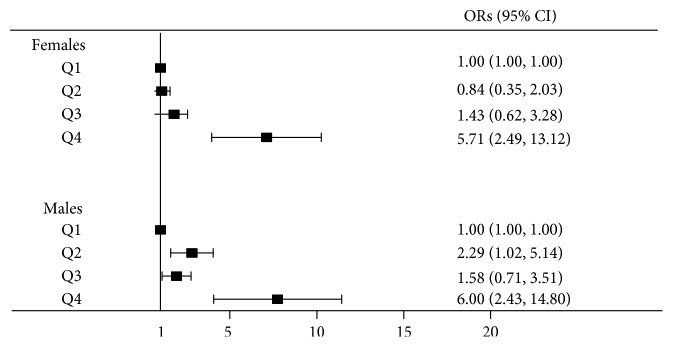
Odds ratios (ORs) and 95% confidence intervals (CI) for *Acanthosis nigricans*, according to serum UA quartiles: results of logistic regression. The ORs (95% CI) for AN in the highest serum UA quartile (Q4) was significantly higher compared to the lowest quartile of serum UA (Q1) for both sexes.

In this study, we used the chi-square tests for trends to compare the prevalence of AN for each quartile among females and males (Table 2). The results showed that the prevalence of AN in Q4 was 66.7% among females and 79.2% among males and was significantly higher compared to Q1 for both men and women (*P* < 0.001, *P* < 0.001, resp.). In logistic regression models, serum UA levels were significantly associated with the prevalence of AN in Q4 in both sexes and the ORs (95% CI) for AN in Q4 were 5.71 (95% CI, 2.49–13.12) among female participants and 6.00 (95% CI, 2.43–14.80) among male participants (Figure 3). Additionally, after adjusting for potential confounders (age and BMI involved in Model 1; age, BMI, FPG, and LDL involved in Model 2; age, BMI, FPG, LDL, and HOMA-IR involved in Model 3), serum UA was still significantly associated with high ORs (95% CI) for AN in both sexes (Table 3). Compared with Q1, among the female participants, the ORs (95% CI) for AN in Q4 were 2.87 (1.10–7.46) in Model 1, 2.91 (1.07–7.91) in Model 2, and 3.00 (1.02–8.84) in Model 3. Among the male participants, the ORs (95% CI) for AN in Q4 were 5.04 (1.88–13.53) in Model 1, 5.67 (2.04–15.75) in Model 2, and 6.07 (2.16–17.07) in Model 3. Moreover, the ORs and 95% CI for AN in male participants in Q2 were significantly higher than those in Q1 in three models.

## 4. Discussion

In our study, we found that a high serum UA level was accompanied with more severe metabolic disorders such as extreme adiposity, hypertension, glucose metabolic disorder, hyperlipidemia, and AN. Moreover, serum UA levels may be an important risk factor for the occurrence of AN independent of BMI, FPG, LDL, and even HOMA-IR.

The association between serum UA and MetS components is in accordance with previous perspective studies. Zhang et al. performed a longitudinal cohort study to explore the relationship between serum UA levels and MetS in Chinese Han urban male population and found serum UA might occur as an important risk factor of MetS [19]. The same results were found in middle-aged Korean men [20] and European individuals [21]. Hyperuricemic animal model caused by fructose-rich diet could induce the components of MetS [22]. Uric acid-lowering drugs (allopurinol or benzbromarone) could blunt the occurrence of MetS, while the rats in the control group developed increased body weight, SBP, hyperinsulinemia, and hypertriglyceridemia. Also, when allopurinol was prescribed, the components of MetS could be prevented [23]. The proposed interlinked mechanisms to explain the relationship between serum UA and MetS apart from insulin resistance were oxidative stress [24], endothelial dysfunction [25], renal microvascular lesions [26], and the imbalance in vasodilation (reduction of nitric oxide [25]) and vasoconstriction (increase of renin-angiotensin-aldosterone-system [19]).

AN is associated with a high prevalence of MetS. In the past years, insulin resistance has been considered as the most important risk factor for AN. The possible explanation was that hyperinsulinemia induced by insulin resistance activated IGF receptors, which were considered to be responsible for mediating the effects of insulin on the proliferation of cells [27, 28], therefore leading to the thickening and hyperpigmentation of the skin of the epidermis and contributing to the development of AN. However, in our study, even when we adjusted the HOMA-IR, we still got a positive relationship between serum UA levels and the occurrence of AN. This indicated that the serum UA levels might be another important factor in the process of AN independent of insulin resistance. We hypothesize that AN prevalence was minimal at Q3 (OR 1.63, 95% CI 0.64–4.19 for UA 459~528 *μ*mol/L in males and OR 0.81, 95% CI 0.28–2.34 for UA 361~424 *μ*mol/L in females) and increased significantly both just below this range at Q2 (2.99, 1.11–8.07 for UA 394~458 *μ*mol/L in males and 0.92, 0.29–2.85 for UA 311~360 *μ*mol/L in females) and throughout this range at Q4 (6.07, 2.16–17.07 for UA ≥ 529 *μ*mol/L in males and 3.00, 1.02–8.84 for UA ≥ 425 *μ*mol/L in females). This relation was just like a U-shaped curve. This is similar to the relationship between BMI and all-cause mortality [29]. The underlying mechanism by which HUA leads to AN may include the following points: firstly, high serum UA induced oxidative stress and increased reactive oxygen species (ROS) levels; secondly, elevated ROS levels subsequently activated phospho-insulin receptor substrate-1 (IRS-1) (Ser307/312) and then inhibited phospho-Akt (Ser473), which finally inhibited the downstream transduction of insulin signaling and led to insulin resistance [30]. However, the relationship between the level of serum UA and AN development was still unclear; further studies were expected to examine the mechanism.

Serum UA is a commonly measured biochemical parameter in health examinations, and our results provide epidemiological evidence that serum UA might be an independent risk factor for the occurrence of AN. Therefore, serum UA level as a serum maker might be used to select obese individuals in order to predict AN and more severe metabolic disorders.

The present study has its limitations. Firstly, in the recent studies, with the fact that the relationship between serum UA levels and metabolic syndrome components was obvious in obese patients instead of normal-weight ones [31, 32], we did not recruit normal-weight patients in our study. Secondly, this study did not assess different lifestyles, dietary habits, and regions, which were found to influence the serum UA levels. Thirdly, this was a cross-sectional study; therefore, it is difficult to ascertain a causal relationship between serum UA levels and occurrence of AN. Finally, the sample size in this study was relatively small which could limit the generalization of our findings. It is also possible that unmeasured confounding variables may exist. Further studies are warranted to elucidate the detailed mechanisms by which high serum UA levels contribute to the prevalence for AN.

In conclusion, our study found that high serum UA levels were associated with more severe metabolic abnormalities including increased BMI, hypertension, hyperglycemia, and hyperlipidemia. Serum uric acid was positively associated with AN and increased the risk of AN. Serum uric acid levels may be a novel and useful method to select obese patients to prevent the occurrence of AN. However, since this study was a cross-sectional study, further evidences in vitro and in vivo are needed to investigate the direct or indirect relationship.

## Conflicts of Interest

The authors declare that there is no conflict of interest regarding the publication of this paper.

## Figures and Tables

**Table 1:** General anthropometric and metabolic characteristics of the study cohort divided by females and males.

Parameters	Female (*n* = 215)			Male (*n* = 196)		
	OB (*n* = 135)	AN (*n* = 80)	*P*	OB (*n* = 85)	AN (*n* = 111)	*P*
UA (*μ*mol/L)	344.2 ± 65.1	407.8 ± 89.1	<0.001^∗∗∗^	430.6 ± 82.4	496.3 ± 112.5	<0.001^∗∗∗^
Age (yr)	31 (12)	26 (12)	<0.001^∗∗∗^	30 (10)	24 (14)	<0.001^∗∗∗^
Weight (kg)	81.77 ± 11.43	95.15 ± 15.23	<0.001^∗∗∗^	106.52 ± 14.79	114.42 ± 20.04	0.002^∗∗^
BMI (kg/m^2^)	30.99 ± 3.52	35.10 ± 5.04	<0.001^∗∗∗^	34.29 ± 3.96	36.79 ± 5.22	0.002^∗∗^
Waist circumference (cm)	99.46 ± 9.49	109.99 ± 12.62	<0.001^∗∗∗^	112.51 ± 10.46	117.03 ± 11.63	0.011^∗^
Waist/hip ratio	0.916 ± 0.069	0.960 ± 0.062	<0.001^∗∗∗^	0.987 ± 0.059	0.998 ± 0.044	0.137
Percentage of body fat (%)	36.98 ± 3.41	38.68 ± 4.38	0.002^∗∗^	31.88 ± 5.21	32.72 ± 4.99	0.940
SBP (mmHg)	128 ± 17	135 ± 15	0.006^∗∗^	133 ± 14	140 ± 14	0.001^∗∗^
DBP (mmHg)	83 ± 10	87 ± 11	0.014^∗^	83 ± 11	85 ± 11	0.305
Cr (*μ*mol/L)	56.88 ± 7.36	56.82 ± 8.88	0.964	74.95 ± 13.71	74.28 ± 13.29	0.760
ALT (U/L)	32.72 ± 26.97	56.96 ± 41.55	<0.001^∗∗∗^	61.73 ± 56.21	85.08 ± 58.03	<0.001^∗∗∗^
AST (U/L)	25.19 ± 14.06	34.77 ± 20.29	<0.001^∗∗∗^	33.92 ± 27.96	43.84 ± 24.35	<0.001^∗∗∗^
TC (mmol/L)	5.02 ± 1.12	4.86 ± 0.93	0.473	4.93 ± 0.95	4.97 ± 1.00	0.774
TG (mmol/L)	1.64 ± 0.99	2.03 ± 2.49	0.399	2.02 ± 1.17	1.79 ± 0.83	0.207
HDL (mmol/L)	1.16 ± 0.26	1.13 ± 0.49	0.056	1.04 ± 0.19	0.97 ± 0.19	0.015^∗^
LDL (mmol/L)	3.08 ± 1.01	2.97 ± 0.86	0.650	3.09 ± 0.83	3.22 ± 0.85	0.307
BG0 (mmol/L)	5.41 ± 1.09	5.77 ± 1.70	0.079	5.81 ± 2.19	5.38 ± 1.10	0.471
INS0 (mU/L)	23.91 ± 21.40	34.12 ± 17.91	<0.001^∗∗∗^	25.64 ± 18.48	39.66 ± 31.74	<0.001^∗∗∗^
CP0 (ng/mL)	3.37 ± 1.28	4.42 ± 1.30	<0.001^∗∗∗^	3.68 ± 1.43	4.75 ± 1.82	<0.001^∗∗∗^
HOMA-IR^a^	1.54 ± 0.64	2.01 ± 0.61	<0.001^∗∗∗^	1.67 ± 0.70	2.04 ± 0.65	<0.001^∗∗∗^

Data are expressed as mean ± SD or median (interquartile range); versus OB, ^∗^*P* < 0.05; ^∗∗^*P* < 0.01; ^∗∗∗^*P* < 0.001; ^a^the data was ln-transformed to normality before analysis. AN: obese group with *Acanthosis nigricans*; OB: simple obese group; BMI: body mass index; SBP: systolic blood pressure; DBP: diastolic blood pressure; UA: uric acid; Cr: serum creatinine; ALT: alanine transaminase; AST: aspertate aminotransferase; TC: total cholesterol; TG: triglyceride; HDL: high-density lipoprotein; LDL: low-density lipoprotein; BG0: 0 min glucose; INS0: 0 min insulin; CP0: 0 min C peptide; HOMA-IR: homeostasis assessment model of insulin resistance.

**Table 2:** Characteristics of participants according to serum UA quartiles in females and males.

Parameters	Quartiles of serum UA in females				*P* for trend
	Q1 (<310)	Q2 (311–360)	Q3 (361–424)	Q4 (≥425)	
*n*	54	53	54	54	
Age (years)	33 (19)	29 (12)	28 (11)	26 (12)	0.001^∗∗^
BMI (kg/m^2^)	31.54 ± 4.64	31.20 ± 4.06	32.74 ± 4.20	34.58 ± 4.77	<0.001^∗∗∗^
Waist circumference (cm)	100.96 ± 13.45	101.63 ± 12.04	103.91 ± 10.18	106.84 ± 11.01	<0.001^∗∗∗^
Hip circumference (cm)	107.89 ± 10.45	109.49 ± 8.62	111.87 ± 9.00	113.68 ± 9.57	<0.001^∗∗∗^
SBP (mmHg)	129.1 ± 15.9	129.6 ± 18.8	131.3 ± 15.6	133.3 ± 16.9	<0.001^∗∗∗^
DBP (mmHg)	82.4 ± 9.3	83.5 ± 12.2	86.4 ± 12.5	87.4 ± 10.7	<0.001^∗∗∗^
TC (mmol/L)	4.84 ± 0.99	4.99 ± 1.25	4.92 ± 0.99	5.13 ± 0.98	0.010^∗^
TG (mmol/L)	1.69 ± 1.06	1.43 ± 0.76	1.66 ± 1.02	2.35 ± 2.95	<0.001^∗∗∗^
HDL (mmol/L)	1.12 ± 0.26	1.15 ± 0.20	1.15 ± 0.28	1.16 ± 0.57	0.001^∗∗^
LDL (mmol/L)	2.99 ± 0.89	3.18 ± 1.05	2.98 ± 0.93	3.03 ± 0.98	0.040^∗^
*Acanthosis nigricans* (%)	25.9	22.6	33.3	66.7	<0.001^∗∗∗^

Parameters	Quartiles of serum UA in males				*P* for trend
	Q1 (<393)	Q2 (394–458)	Q3 (459–528)	Q4 (≥529)	

*n*	49	49	50	48	
Age (years)	28 (13)	30 (13)	27 (12)	23 (15)	0.098
BMI (kg/m^2^)	34.39 ± 4.81	35.02 ± 4.22	36.32 ± 4.57	37.12 ± 5.48	<0.001^∗∗∗^
Waist circumference (cm)	112.48 ± 12.87	113.28 ± 9.58	115.41 ± 9.88	119.19 ± 11.86	<0.001^∗∗∗^
Hip circumference (cm)	114.26 ± 9.22	113.10 ± 7.04	115.69 ± 8.69	120.46 ± 11.42	<0.001^∗∗∗^
SBP (mmHg)	135.4 ± 14.2	135.2 ± 14.5	135.4 ± 14.1	142.1 ± 15.8	0.005^∗∗^
DBP (mmHg)	83.9 ± 10.3	85.0 ± 10.9	85.2 ± 10.7	84.4 ± 12.8	0.912
TC (mmol/L)	4.72 ± 0.83	5.18 ± 1.11	5.06 ± 1.03	4.86 ± 0.87	0.359
TG (mmol/L)	1.67 ± 0.93	1.92 ± 1.03	2.04 ± 1.05	1.95 ± 0.97	0.256
HDL (mmol/L)	1.05 ± 0.19	1.00 ± 0.16	1.02 ± 0.23	0.95 ± 0.19	0.005^∗∗^
LDL (mmol/L)	2.95 ± 0.69	3.37 ± 0.96	3.23 ± 0.89	3.09 ± 0.76	0.186
*Acanthosis nigricans* (%)	38.8	59.2	50	79.2	<0.001^∗∗∗^

*P* value by linear regression analysis and chi-square tests for trends in proportions. ^∗^*P* < 0.05; ^∗∗^*P* < 0.01; ^∗∗∗^*P* < 0.001.

**Table 3:** Odds ratios (ORs) and 95% confidence intervals (CI) for AN, according to serum UA quartiles: results of binary logistic regression analysis in different models.

Models	Independent variables	*P*	OR (95% CI)	Independent variables	*P*	OR (95% CI)
	Female			Male		
Model 1	Q1	0.017^∗^	1.00 (reference)	Q1	0.005^∗∗^	1.00 (reference)
	Q2	0.603	0.77 (0.28–2.09)	Q2	0.014^∗^	3.07 (1.25–7.53)
	Q3	0.782	0.87 (0.33–2.28)	Q3	0.369	1.48 (0.63–3.50)
	Q4	0.031^∗^	2.87 (1.10–7.46)	Q4	0.001^∗∗^	5.04 (1.88–13.53)

Model 2	Q1	0.036^∗^	1.00 (reference)	Q1	0.004^∗∗^	1.00 (reference)
	Q2	0.823	0.89 (0.31–2.54)	Q2	0.017^∗^	3.22 (1.23–8.42)
	Q3	0.909	0.94 (0.35–2.54)	Q3	0.413	1.47 (0.59–3.66)
	Q4	0.036^∗^	2.91 (1.07–7.91)	Q4	0.001^∗∗^	5.67 (2.04–15.75)

Model 3	Q1	0.030^∗^	1.00 (reference)	Q1	0.004^∗∗^	1.00 (reference)
	Q2	0.889	0.92 (0.29–2.85)	Q2	0.030^∗^	2.99 (1.11–8.07)
	Q3	0.710	0.81 (0.28–2.34)	Q3	0.308	1.63 (0.64–4.19)
	Q4	0.046^∗^	3.00 (1.02–8.84)	Q4	0.001^∗∗^	6.07 (2.16–17.07)

^∗^ *P* < 0.05; ^∗∗^*P* < 0.01; Model 1: age and BMI were selected. Model 2: age, BMI, FPG, and LDL were selected. Model 3: age, BMI, FPG, LDL, and HOMA-IR were selected.
